# Different clinical diagnosis end up in the same pathological diagnosis of intravascular leiomyomatosis: Two case reports

**DOI:** 10.1097/MD.0000000000036887

**Published:** 2024-01-12

**Authors:** Yang Tan, Jing Han, Zhenglan Wang, Ju Yan, Lin Dong, Rui Liu

**Affiliations:** a Department of Pathology, Dujiangyan Maternity and Child Health Hospital, Chengdu, Sichuan, China; b Department of Pathology, Huayin Medical Laboratory Co., Ltd, Chengdu, Sichuan, China.

**Keywords:** diverticula, fibroid, hysterectomy, intravascular leiomyomatosis, intravenous leiomyomatosis, perimenopause

## Abstract

**Rationale:**

Intravascular/intravenous leiomyomatosis (IVL) is a peculiar variant of uterine leiomyoma that is classified as a histologically benign smooth muscle tumor with a biological behavior similar to that of a malignant tumor. It is characterized by the proliferation of leiomyomas spreading along the uterine and extrauterine venous circulation.

**Patient concerns:**

Herein, we present 2 cases of IVL who had completely different clinical manifestations to stress the need for constant vigilance of IVL diagnosis and the understanding of uterine leiomyoma heterogenicity. Case 1 was registered for fever without specific triggering factors, irregular menstruation and clinically diagnosed uterine diverticula, while no information about fibroids was mentioned. Case 2 was characterized by an aggressively growing abdominal mass. With a large space-occupying lesion in the right abdominopelvic cavity and no imaging evidence of involvement of the iliac vein or above vein, the patient was initially diagnosed with multiple myomata.

**Diagnoses:**

Both patients’ diagnoses were confirmed as IVL by histopathology. To our knowledge, the mass of case 1 is the minimum IVL in the English literature.

**Interventions:**

Subtotal hysterectomy with bilateral salpingectomy was performed on the former, while total hysterectomy with bilateral salpingectomy was performed on the latter.

**Outcomes:**

Both patients were comfortable, and no relapse occurred.

**Lessons:**

Two cases in the study showed 2 different proceeding stages of the same disease and corroborated multiple pathogeneses, which have been mentioned in the available literature on IVL. Our work provides both supplement for clinical data to facilitate further research and better understanding of special types of fibroids to clinicians.

## 1. Introduction

Intravascular/intravenous leiomyomatosis (IVL) is a relatively rare tumor that is histologically benign but potentially extends to vascular tunnels from the intrauterine venules upwards to varying distances.^[1]^ It is one variant of uterine fibroid. The term “Leiomyoma with vascular invasion” is used when intravenous foci are confined to hysteromyoma and have no risk of relapse.^[2]^ Most patients are usually asymptomatic in an early stage, but once the cardiovascular system is involved the clinical presentation is variable and mostly related to the severity of cardiac involvement. Surgery is the main treatment, and the surgical approach varies in patients with different clinical stages.^[3,4]^ Some scholars believe that IVLs are hormone-dependent tumors, while others verify that hormonal therapies are useless, which indicates that ovaries preservation is feasible.^[4–6]^ In this treatise, we present 2 cases to exhibit the stealth and heterogenicity of IVL. One is a 45-year-old woman who was referred to our hospital with successively emerged menoxenia, fever, and diverticula and was eventually demonstrated to have the minimum IVL to our knowledge. The other patient was a 53-year-old woman who was registered for abdominal mass and clinically suspected uterine leiomyoma. What was unusual of our first patient was the absence of any hints of uterine myoma in abdominal and pelvic ultrasound and magnetic resonance imaging (MRI) examination. In addition, the intravenous filling defect of the serosal surface was neglected in the second patient. Clinicians and radiologists should have a high index of vigilance for IVL diagnosis. Our work may deepen the understanding of IVL diagnosis and provide supplemental clinical data to facilitate further research.

## 2. Case presentation

### 2.1. Case 1

A 45-year-old woman, gravida 1, para 1, was admitted to the gynecology department for 5 days’ fever without notable incentive; by then, she had suffered menometrorrhagia for a 45-day period. Intrauterine infection was initially considered. A month prior to admission, she had taken a progesterone capsule 20 mg daily to treat irregular bleeding, which worked less well. Her past medical history was remarkable for a cesarean 19 years ago and a transabdominal oophorocystectomy 7 years ago.

**Figure 1.**
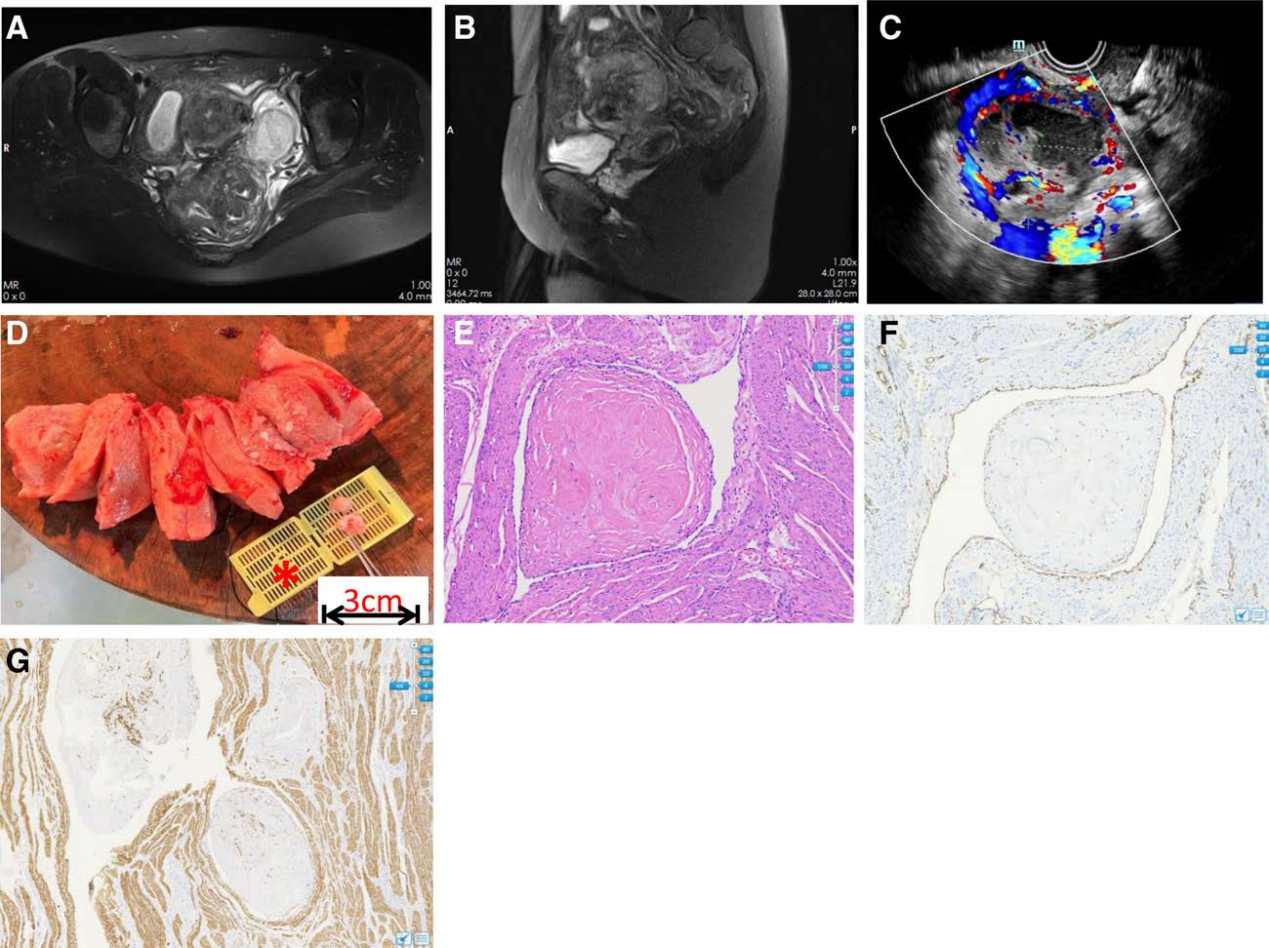
The clinical and pathological features of case 1. (A and B) Magnetic resonance imaging (MRI) shows a diverticular-like appearance (approximately 6 cm in diameter) in the anterior uterine wall and a cyst measuring 1.8 cm in diameter in the diverticular wall. (C) Abdominal and pelvic ultrasonography suggested space-occupying lesions with diffuse vascular cavity structure in the uterus wall, and a rich blood supply was observed. (D) The macroscopic appearance shows some white, rubbery, worm-like intravascular masses springing up out of the vascular tunnel of the uterus (scale bar refers to embedded box length*). (E) Intravascular tumors filling the venous lumen were stained with hematoxylin and eosin (H&E). Typical smooth muscle cells. The spindle cells were arranged in an interlaced shape with no obvious atypia. (F) Immunohistochemical (IHC) staining for the CD31 marker, confirming that the tumor mass grew within endothelial vascular walls. (G) The tumor is positive for Desmin marker, confirming the component of smooth muscle in IVL.

Nothing special about physical examination was found. She underwent abdominal and pelvic ultrasound (Mindray, China) and magnetic resonance imaging (MRI, Siemens, Germany) examinations in the outpatient department. Blood test findings after admission revealed bacterial infection with a rising differential count of neutrophils to 75.3% (reference range in our laboratory is 50–70); however, these findings were otherwise unremarkable. Abdominal and pelvic ultrasound demonstrated a mixed echo area (measured 6.4 cm × 4.8 cm) of the left adnexa uteri and a medium echo area (measured 4.1 cm × 3.8 cm) of the right adnexa uteri. MRI was strongly suggestive of a diverticula-like appearance in the scar of the anterior uterine wall and was approximately 6 cm in diameter. There was an uneven signal within the diverticulum, which was a cue for infection. A cyst, approximately 1.8 cm in diameter, was visible in the diverticular wall. There was an irregular thick-wall cystic space-occupying lesion in the left appendage region with a range of approximately 6.2 cm × 4.8 cm (Fig. 1). Given the workup findings and symptoms, the main diagnosis of diverticula and endometriosis had been made. A subtotal hysterectomy and bilateral salpingectomy were performed.

**Figure 2.**
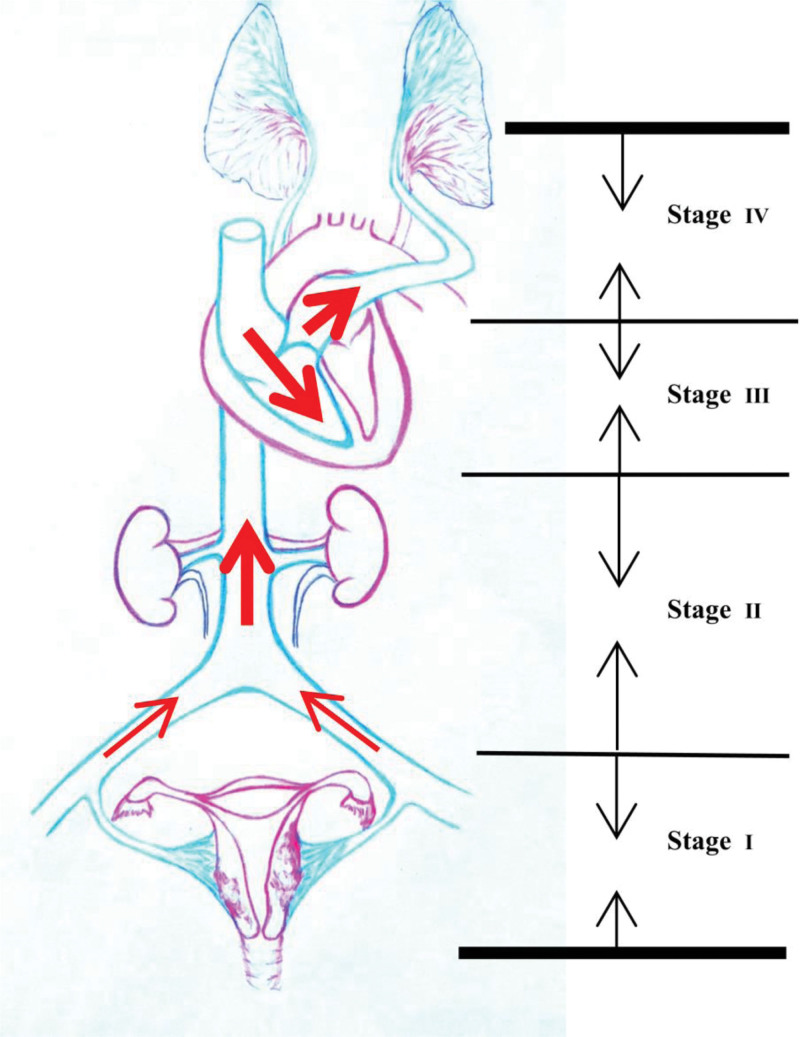
The sketch map of the clinical staging system. In stage I, the tumor is confined to the pelvis. In stage II, the tumor is located in the iliac vein/inferior vena cava. In stage III, the tumor entered the right atrium/right ventricle. In stage IV, the tumor reached the pulmonary artery.

Some smooth, white, worm-like intravascular masses sprung up out of the vascular tunnel of the uterine cut surface postoperatively (Fig. 1). The maximum diameter of the lesion composed of an isolated leiomyoma and scattered intravascular foci was approximately 1.7 cm. There was no tight adhesion between the tumor tissue and the tube wall, which resulted in easy separation and peeling. Microscopic examination revealed spindle-shaped smooth muscle cells with eosinotropic cytoplasm and cigar-like nuclei woven into a bundle-like arrangement. The tumor was partially located in the vascular vessels and was enwrapped by endothelial cells, while obvious hyaline degeneration in the stroma was observed (Fig. 1). There were no atypical spindle cells, rare mitosis, and no necrosis. Immunohistochemical staining (Sigma-Aldrich, Germany) was positive for smooth muscle actin, desmin, estrogen receptor, and progesterone receptor. Focal positive CD10 expression was perceived. The endotheliocytes diffusely expressed CD31 and ETS-1 related gene (Fig. 1). The Ki-67 index is approximately 1%, which is concordant with previous reports of usually <5%.^[6]^ IVL was not the sole lesion in this patient, and concomitant gynecological disorders included adenomyosis, diverticula, and chronic suppurative inflammation of the diverticula and bilateral fallopian tubes. This tumor confined to the uterus was in stage I according to the clinical staging system (Fig. 2).^[7]^

In clinical practice, the disease should be differentiated from vascular leiomyoma, low-grade malignant endometrial stromal sarcoma, leiomyosarcoma with vascular invasion, and dissecting leiomyoma. Nine days postoperatively, the patient was discharged home without any complications and was followed up every 3 months, and no recurrence was noticed.

The timeline of case 1 is shown in Table 1.

**Table 1:** The key timeline of clinical course of case 1.

Phrase	Clinical event	Day −45	Day −30	Day −5	Day −4	Day −2	Day 0	Day 1	Day 4	Day 14	Day 90	Day 120
Preoperative	Irregular bleeding of initial symptom											
	Taken progesterone capsule 20 mg daily to treat menorrhagia											
	Got fever without specific triggering factor											
	Ultrasonography found space-occupying lesions in bilateral adnexal area											
	MRI displayed a diverticular-like appearance with a cyst in its wall (Fig. 1A)											
	Registered in our hospital for further treatment											
Intraoperative	Underwent surgery of subtotal hysterectomy with bilateral salpingectomy (Fig. 1)											
Postoperative	Recovered well without complication and discharged home											
	Immunohistochemical staining combining with paraffin section confirmed the diagnosis of IVL (Fig. 1)											
	Surveillance CT scan of the abdomen and pelvis show no evidence of recurrence											
	As of submission, the patient felt well by telephone follow-up											

CT = computed tomography scan, IVL = intravascular/intravenous leiomyomatosis, MRI = magnetic resonance imaging.

### 2.2. Case 2

**Figure 3.**
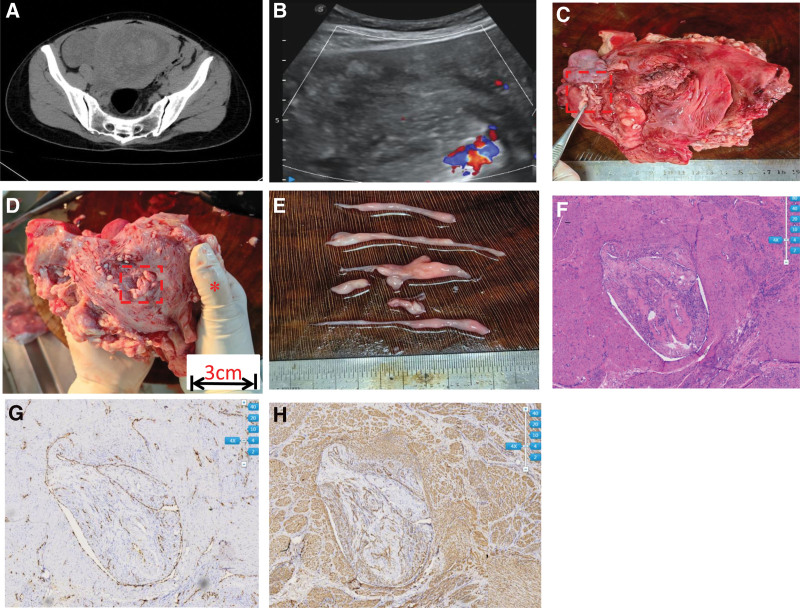
The clinical and pathological features of case 2. (A) Computed tomography (CT) scans of case 2: a CT scan demonstrated a giant heterogeneous space-occupying lesion in the right abdominopelvic cavity (13.8 × 5.7 cm) with mixed mottling and patchy heterogeneous density. (B) Abdominal and pelvic ultrasonography revealed a large intrauterine mass in the anterior wall of the uterus and a large cord-like mass in the right broad ligament. (C and D) The gross specimen presented the myometrium and serous membrane intravenous masses separately (scale bar refers to finger width*). (E) The snakelike tumors, removed from the C,D box, were 0.7 cm to 4 cm in length and 0.05 cm to 0.6 cm in diameter. (F) Intravenous filling defects with hematoxylin and eosin (H&E) staining revealed characteristics of both epithelioid leiomyoma and ordinary leiomyoma. (G) Immunohistochemical (IHC) staining for ETS-1 related gene (ERG) markers, confirming that the tumor mass grew within endothelial vascular walls. (H) The tumor is positive for smooth muscle actin (SMA) markers, confirming the component of smooth muscle in IVL. IVL = intravascular/intravenous leiomyomatosis.

A 53-year-old menopausal woman, gravida 2, para 2, without any past medical history or family history of cancer, was registered for gradually increased abdominal distension. A 2 cm fibroid was detected in a routine checkup 2 years ago, and regular gynecological surveillance was advised. She found an abdominal bulge by self-palpation 3 months earlier and was aware of its apparent increase 4 days prior to admission. The patient was comfortable with vital signs within normal limits. Initial laboratory studies were not noticeable. Ultrasonography revealed intrauterine and middle right abdominal masses measuring 10.5 × 6.9 × 9.7 cm and 12.2 × 6.1 × 8.1 cm, respectively. A computed tomography scan (Siemens, Germany) demonstrated a giant heterogeneous space-occupying lesion in the right abdominopelvic cavity (13.8 × 5.7 cm) with mixed mottling and patchy heterogeneous density (Fig. 3). Given the blurry findings mentioned above, the main diagnosis was hysteromyoma. Consequent total abdominal hysterectomy with bilateral salpingectomy was conducted. During laparotomy, the anterior wall of the unevenly enlarged uterus partially adhered to the omentum majus. A myometrial mass (diameter 11 cm) as well as a rope-like mass (18 cm × 10 cm × 3.8 cm) in the right broad ligament were found.

The macroscopic appearance showed a dissociative cord-like mass, an intrauterine mass, and several distensible veins on the serous membrane within snakelike filling lesions, which had a rubber-like texture and could be easily drawn out from channels (Fig. 3). Near the myometrial mass (at a distance of 1.5 cm), there was a worm-like region measuring 3 cm × 1.5 cm × 1 cm, which was just underneath the serosal vascular defects (Fig. 3). The microscopic appearance of intra- and extravascular lesions presented the characteristics of epithelioid leiomyoma and ordinary leiomyoma. Hyaline degeneration combined with hydropic degeneration was distributed in the mesenchyme. No atypia, no mitosis, and a very low Ki-67 index of approximately 3% were observed. Immunohistochemical staining was positive for smooth muscle cell markers (desmin and smooth muscle actin) and peritumor vascular endothelial markers (CD31 and ETS-1 related gene) (Fig. 3).

This tumor, beyond the uterus and limited to the pelvis, was in stage I according to the clinical staging system (Fig. 2).^[7]^ Hence, no supplementary therapy was applied after surgery. The patient was discharged from admission after an uncomplicated postoperative course of 8 days. She was reviewed at 1, 3, and 6 months and annually afterwards, and no abnormalities were noted to date. The timeline of case 2 is shown in Table 2.

**Table 2:** The key timeline of clinical course of case 2.

Phrase	Clinical event	2020	Day −90	Day −4	Day 0	Day 1	Day 3	Day 6	Day 8	Day 13	Day 30	Day 90
Preoperative	A 2 cm fibroid was detected by her primary medical examination.											
	Abdominal distension was found by self-palpation.											
	The patient sensed the gradually increased size of abdominal mass.											
	The patient sought medical attention at our hospital and hospitalized.											
	Ultrasonography and CT scan revealed a large intrauterine mass of the anterior wall of uterus and a huge cord-like mass in the right broad ligament(Fig. 2).											
	Underwent surgery of total abdominal hysterectomy with bilateral salpingectomy.											
Intraoperative	Preliminary pathological findings hinted microscopic features of IVL (Fig. 2).											
Postoperative	The patient discharge from admission.											
	Immunohistochemical findings combining with paraffin section confirmed the diagnosis of IVL (Fig. 2).											
	Surveillance CT scan of the abdomen and pelvis showed in a good clinical condition.											
	The general condition was quite good in the second follow-up of CT scan.											

CT = computed tomography scan, IVL = intravascular/intravenous leiomyomatosis.

## 3. Discussion

IVL is a histologically benign tumor that grows within veins and rarely lymphatic vessels but does not invade the circumambient tissue.^[8]^ It often affects perimenopausal women with variable and nonspecific symptoms such as menstrual changes, pelvic mass, phlebothrombosis, or a right atrial mass depending on the progression of the entity. Herein, we present 2 IVL cases with completely different clinical presentations: one was verified to have minimum IVL in the current literature, and the other was stage I with large intrauterine and broad ligament masses.

The incidence is low, but the recurrence rate postoperatively is very high.^[9–11]^ Approximately 400 cases have been reported in the English literature since it first appeared in 1896.^[10]^ Since a preoperative definite diagnosis could not be made by virtue of clinical symptoms and imaging analysis, the number of IVL cases remains underestimated due to its relatively insidious onset.^[11–13]^ Following reasons such as asymptomatic IVL patients, undiagnosed early IVL patients, unreported limited lesions, etc, cause the morbidity to be underrated. Case 1, which was undiagnosed preoperatively, turned out to be an incidental IVL patient. The widespread application of minimally invasive surgery makes pathological characteristics atypical, which also leads to the underdiagnosis of IVL. The literature reports that the recurrence rate is approximately 16.6% to 30%.^[4,8]^ Following factors such as the degree of tumor resection, patients with large lesions (≥7 cm) and lesions extending to the broad ligament were deemed to be closely associated with recurrence.^[6]^ There is little dispute that surgical type was the most important high-risk factor. The tumor should be removed as completely as possible regardless of the first operation or the recurrence condition. No significant difference in the recurrence rate was discovered between total hysterectomy (TH) patients and total hysterectomy with bilateral oophorectomy (TH-BSO) patients. The patients with tumorectomy had a 20 times higher recurrence rate than those with TH and TH-BSO.^[14]^ Both of our patients retained ovaries that coincided with the pertinent literature’s view, and they were kept under constant surveillance. Recent studies also revealed that younger patients (age ≤ 45 years) were more prone to recurrence, which needs more research to confirm.^[12]^

A clinical staging system was proposed by Ma et al^[7]^ in 2016 to reflect the preoperative progression of IVL and help formulate a therapeutic regimen. Stage I: lesions were confined to the pelvis; stage II: lesions were located in the iliac vein/inferior vena cava; stage III: tumors entered the right atrium/right ventricle; stage IV: tumors reached the pulmonary artery.^[7]^ For early-stage patients, complete tumor excision is the key to obtaining a favorable prognosis. However, it is a challenge to choose an appropriate surgery strategy when it is headed to advanced stages due to the lack of corresponding guidelines. One-stage surgery and 2-stage surgery are optional according to patient conditions, and multidisciplinary cooperation is requisite in complex situations.^[11]^ However, the size of IVL ranged from approximately 1.7 cm to 15.5 cm, and both of our patients were in stage I. To our knowledge, the size of IVL in case 1 is minimal according to the available English literature. Some reports have postulated that the larger the mass is, the more likely it is to involve the cardiovascular system.^[11]^ Patients with large lesions (≥7 cm) and lesions extending to the broad ligament may have an increased risk of recurrence, and parauterine metastasis is the first step toward extrapelvic involvement.^[6,14]^ Although the mass filling abdominopelvic cavities of case 2 was very large, it was just expansive growth with no imaging evidence of iliac vein or inferior vena cava involvement. We speculate that the reason is the short course of the enlarging process. To be prudent, the patient was informed to review at 1, 3, and 6 months and annually afterwards.

The clinical curative effect of hormonal adjuvant therapy is mutually contradictory. Su et al^[12]^ proposed that if a definitive diagnosis is made during surgery, the uterus and bilateral attachments, including the tumor, should be removed. In addition, the ovary and uterine vein should be ligated as high as possible. However, other scholars believe that neither ovarian resection nor postoperative hormonal therapy is associated with recurrence. Some compromised that young patients, such as those aged approximately 40 years, BSO, and postsurgical GnRH-a hormonal therapy, may be considered, but whether these methods can obtain a better prognosis needs long-term studies.^[4,12,15,16]^ None of our patients accepted postsurgical hormonal therapy, and both had good clinical conditions, as in this report.

When the tumor is confined to the uterus and pelvic cavity, an accurate diagnosis of IVL is difficult to make preoperatively. While the lesions extend to vessel lumens or even to the chambers of the right heart, following differential diagnosis as right atrial myxoma, renal carcinoma, intravenous leiomyoma sarcoma, and DVT, should be considered. Although preoperative imaging examination helps little to distinguish IVLs from ordinary leiomyomas unless the disease has processed into an advantage stage, these techniques aid in revealing the relationship between venous masses and pelvic masses, which is helpful in determining the extent of uterine IVL. Some scholars believe that contrast-enhanced ultrasound has higher diagnostic accuracy, while others tend to recommend advanced chest and abdominal computed tomography scan, gynecological ultrasound, and MRI if necessary as better surveillance methods according to different clinical conditions.^[12,13]^ Both of our patients were initially diagnosed with unspecified pelvic space-occupying lesions by ultrasound, and MRI referred to only diverticula, endometriosis, and inflammation in case 1. This urges radiologists to enhance the thorough understanding of IVL’s imaging diagnosis. Histopathology is considered to be the gold standard to diagnose IVL. Sampling and embedding in appropriate measures is a real headache for pathologists because the intravascular mass can easily retract or protrude from the vein. If the section does not include the tumor, surrounding normal muscle tissues and intravenous lesions, misdiagnosis, and underdiagnosis may occur. Lobulated and slit-shaped tumors can be seen under the microscope, and the morphology manifested as cellular leiomyoma, epithelioid leiomyoma, myxoid leiomyoma, lipoleiomyoma, and leiomyoma with bizarre nuclei. The histopathology features of case 1 were the same as those of ordinary leiomyomas, while those of case 2 appeared as both epithelioid leiomyomas and classical myomas. Hyaline degeneration and hydropic degeneration in the stroma, similar to our cases, are present in many patients.^[2]^

The exact pathogenesis of IVL remains to be elucidated, and to date, there are 2 main hypotheses about its origin. The first one assumed that the tumor originates from the smooth muscle layer of the blood vessel wall, while the other one postulated that IVLs are the result of myoma tissue infiltrating into the uterus or parauterine veins. The cases described above seem to favor the latter hypothesis. There are still other hypotheses, such as some researchers who have proposed that IVL might originate from the myometrium rather than fibroids.^[6]^ In view of most patients with IVL having a history of uterine operation, some studies have provided the possibility that local injuries and vascular damage due to surgeries may create conditions for the genesis and development of this disease. Factors including elevated estrogen levels, venous blood stasis, and intrauterine device placement have all been considered contributors to the onset of IVL.^[10,14]^ The abovementioned results suggest that IVL might be caused by the cooperation of multiple factors. For example, our 2 cases were registered for completely different clinical manifestations, different imaging and laboratory examination findings, different medical histories, different natural courses, and different macroscopic appearances, demonstrating the diversity of IVL clinicopathologic features and pathogenesis.

Although the number of studies, especially case reports, on IVL has been increasing in recent years, this disease still lacks attention from clinicians. It is necessary for radiologists, pathologists, and surgeons to obtain a better understanding of IVL and to be on their guard in clinical practice. The status quo demands collecting more raw clinical data for further research to form systematic guidelines for the diagnosis and treatment of patients with IVL.

Due to the limitations inherent to case reports, this study had some limitations. First, this is the first 2 cases of IVL in the last 12 years since the establishment of the pathology department of our hospital, and we have little experience in diagnosis and clinical arrangement. Second, the small sample size cannot form a systematic study of the clinical and pathological features of this rare disease. Last, the patients had short-term follow-up, and the long-term prognosis was ambiguous.

The teaching points are as follows. Intravascular leiomyomatosis is a rare, benign muscular cell tumor with intraluminal growth and extension. The nonspecific clinical manifestation makes it hard to accurately diagnose unless the tumor has invaded the inferior vena cava or above with serious clinical symptoms. Given the high recurrence, patients should be vigorously followed. Clinicians should know special types of fibroids in clinical practice.

## Author contributions

**Data curation:** Yang Tan, Zhenglan Wang, Ju Yan.

**Methodology:** Jing Han, Zhenglan Wang, Ju Yan.

**Project administration:** Lin Dong.

**Resources:** Jing Han.

**Writing – original draft:** Yang Tan.

**Writing – review & editing:** Lin Dong, Rui Liu.
